# Spontaneous regression of multiple flow-related aneurysms following treatment of an associated brain arteriovenous malformation: A case report

**DOI:** 10.3389/fsurg.2022.860416

**Published:** 2022-12-16

**Authors:** Lukas Andereggen, Philipp Gruber, Javier Anon, Angelo Tortora, Hans-Jakob Steiger, Gerrit A. Schubert, Serge Marbacher, Luca Remonda

**Affiliations:** ^1^ Department of Neurosurgery, Kantonsspital Aarau, Aarau, Switzerland; ^2^ Faculty of Medicine, University of Bern, Bern, Switzerland; ^3^ Department of Neuroradiology, Kantonsspital Aarau, Aarau, Switzerland

**Keywords:** intracranial aneurysm, arteriovenous malformation, subarachnoid hemorrhage, angiography, surgery

## Abstract

**Introduction:**

There is no consensus in the treatment strategy of intracranial aneurysms (IAs) associated with brain arteriovenous malformation (BAVM). In particular, it is unknown if a more aggressive approach should be considered in patients harboring a BAVM, in whom multiple aneurysms or a history of aneurysmal subarachnoid hemorrhage (aSAH) is present.

**Case presentation:**

We report on an elderly woman harboring multiple aneurysms with a history of SAH due to rupture of an unrelated IA. On evaluation, she was also found to harbor a contralateral, left parietal convexity BAVM. Following resection of the latter, spontaneous regression of two large flow-related aneurysms was encountered.

**Discussion:**

We discuss the therapeutic decision-making, risk stratification, and functional outcome in this patient with regard to the pertinent literature on the risk of hemorrhage in IAs associated with BAVM.

## Introduction

Intracranial aneurysms (IAs) in patients with brain arteriovenous malformation (BAVM) can be encountered on unrelated vessels or associated with the BAVM and classified as proximal, intranidal, or distal flow related (1, 2).

The need for treatment of flow-related IAs associated with BAVM is controversial. While flow-related IAs potentially harbor a higher risk of intracerebral hemorrhage (ICH), thus entailing a more unfavorable natural history (3), there is no consensus on its treatment strategy. Treatment of proximal flow–related IAs has been favored given the risk of rupture when sudden hemodynamic changes at the time of BAVM elimination occur (4), potential flow reduction through feeding arteries and subsequent IA regression following BAVM extirpation have prompted others to suggest the elimination of the BAVM first (5, 6). Thus, risk assessment on the presence of flow-related IAs in BAVM remains difficult, and no recommendation in these patients exists. Furthermore, it is unknown if a more aggressive approach should be considered in high-risk patients who are harboring multiple aneurysms or have a history of aneurysmal subarachnoid hemorrhage (aSAH).

Here, we report on an elderly woman with BAVM and multiple associated IAs, who had a history of aSAH due to rupture of an unrelated IA. Spontaneous regression of two flow-related aneurysms was encountered on complete resection of the associated BAVM. We discuss the therapeutic decision-making and risk stratification with regard to the pertinent literature on IAs associated with BAVM.

## Case presentation

**Figure 1:**
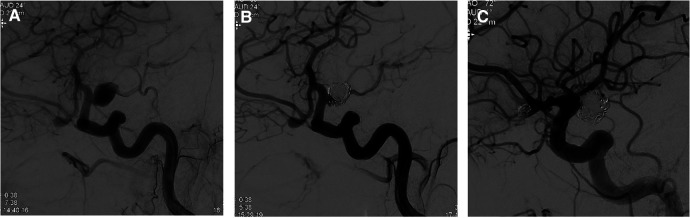
DSA at baseline and at 6 months’ follow-up. (**A,B**) Sagittal angiogram showing coil embolization of a ruptured PComA on the right side. (**C**) Coil embolization of the unruptured AComA aneurysm at 6 months’ follow-up without missing sings of PComA reperfusion (both MRRC, class II).

**Figure 2:**
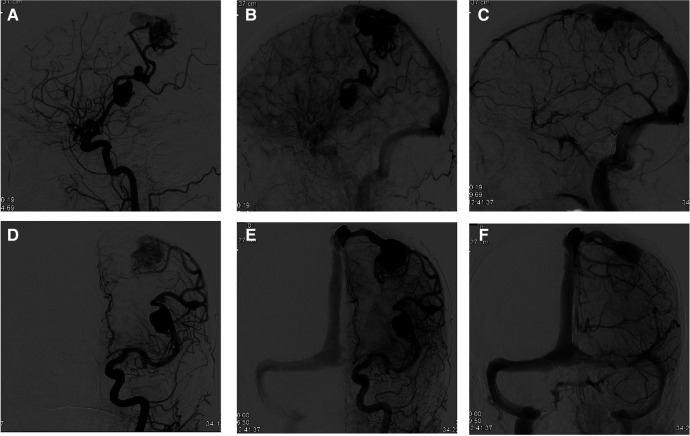
Early, middle, and late phases of DSA. **(A–C**) Sagittal (lateral) and (**D–F**) coronal (anterior-posterior) angiograms (views) demonstrating the adjacent flow-related MCA aneurysms on the left side with the associated BAVM. Note the presence of the BAVM venous ectasia in the later phase (**C,F**).

**Figure 3:**
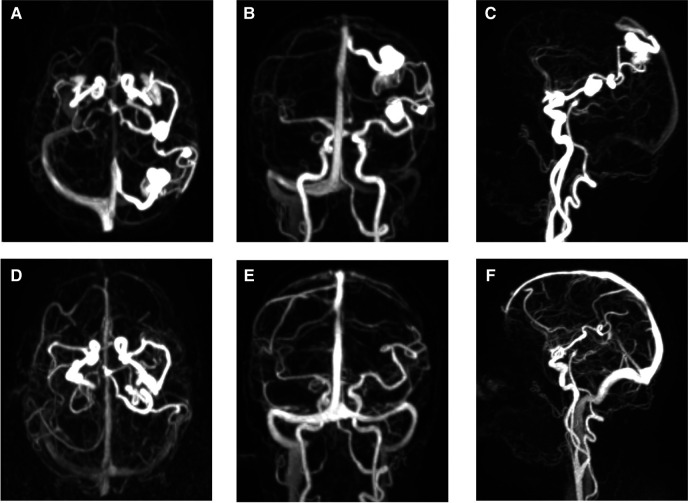
TWIST MR angiography before and after BAVM elimination. (**A–C**) Axial, coronal, and sagittal TWIST (time-resolved angiography with interleaved stochastic trajectories) MR angiography depicting both flow-related MCA aneurysms and associated BAVM. (**D–F**) Complete regression of the flow-related MCA aneurysms following the encounter of BAVM elimination.

**Figure 4:**
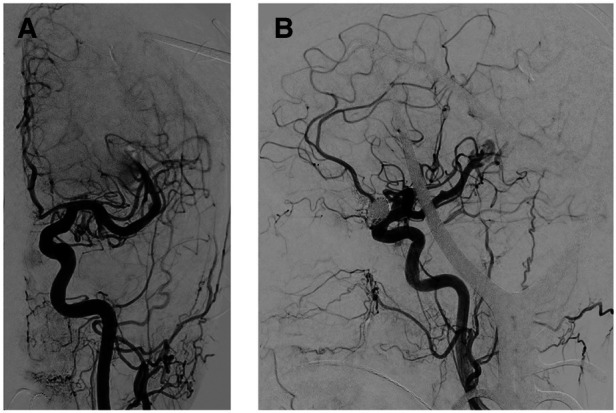
Intraoperative angiogram after BAVM elimination. (**A,B**) Coronal and sagittal angiogram depicting regression of the flow-related MCA aneurysms following the encounter of BAVM elimination.

A 66-year-old woman suffered from aSAH due to rupture of a 10 mm posterior communicating artery aneurysm (PComA) on the right side. The three-dimensional digital subtraction angiography (3D-DSA) further demonstrated a 4 mm anterior communicating artery aneurysm (AComA) that filled arteriographically from the right side (Figure 1). In addition, we encountered a left postcentral gyral neopallial BAVM (Supplemented Spetzler-Martin, SSM, grade 5; namely size 1 point, venous drainage 0 points, eloquence 0 points, age 3 points, bleeding 1 point, compactness 0 points) (7), with two large distal flow–related aneurysms of the middle cerebral artery (MCA) on the M2 segment (20 mm) and the M3 segment (8 mm), (Figure 2) respectively. The ruptured PComA was coil embolized (Modified Raymond-Roy Classification, MRRC, class II) (8) and the patient harbored an uneventful recovery from the aSAH (Glasgow Outcome Scale, GOS 5). Six months after the ictus, the AcomA was coil embolized. One year after aSAH, interdisciplinary decision-making was consented for combined endovascular and surgical elimination of the BAVM before treatment of the flow-related IAs (7). The BAVM was intraoperatively partially embolized with Onyx (Medtronic, Minneapolis, United States) using a transarterial approach, and then surgically extirpated. Both indocyanine green (8) and intraoperative 3D-DSA (9–11) revealed complete elimination of the BAVM, along with early regression of both flow-related MCA aneurysms (Figures 3, 4). One-year follow-up was uneventful, with no evidence of reperfusion of the flow-related MCA aneurysms on MR angiography.

## Discussion

Risk assessment in the presence of flow-related IAs in BAVM is difficult as it is mostly based on case series and, as such, represents Class III evidence (12). In the randomised trial of Unruptured Brain Arteriovenous malformations (ARUBA), the incidence of AVM-associated (i.e., flow-related and located on an AVM feeding artery or intranidal) and unrelated IAs was 16.1% and 4.9%, respectively (13). While information on spontaneous regression of IAs upon AVM treatment was not particularly reported, Gross and Du calculated in their meta-analysis that the presence of IAs increased the risk of hemorrhagic presentation by a factor of 1.8 (14). Brown RD et al. reported that the risk of hemorrhage among patients with IAs and a coexisting unruptured AVM was 7% at 1 year compared with 3% among those with an AVM alone (15). Certain authors have suggested that prenidal IAs are more likely to present with hemorrhage compared with intranidal aneurysms (5, 16), while others have found that distal flow-related and intranidal aneurysms that are immediately adjacent to the site of arteriovenous shunting may be more prone to rupture, given the higher flow, pressure, and shear stress on the vessel wall (17), with yet inconsistent results (18). As such, the need for treatment of flow-related IAs associated with BAVM is an ongoing matter of debate, as treatment strategy remains empirical. In addition, it is unknown if a more aggressive approach should be considered in patients who are harboring multiple aneurysms or present with history of aneurysmal aSAH. Given the missing treatment guidelines, the treatment risk should be carefully weighed against the natural history of the disease, which remains poorly understood (14). On one hand, it has been proposed that treatment of associated IAs in unruptured BAVMs should follow the same guidelines that exist for the treatment of unruptured IAs (19). On the other hand, IAs are generally thought to harbor an increased risk of hemorrhage when being associated with BAVMs (3–5, 14, 20). In contrast, unrelated IAs do not appear to increase the risk of BAVM rupture, carrying similar risk of hemorrhage to that of common saccular aneurysms (19).

Given the size of the flow-related IAs, treatment might have been indicted with regard to potential rupture before the elimination of the BAVM. It can be argued that given the history of aSAH, treatment toward direct endovascular coil embolization is favored (21, 22). The use of adjuvant devices for assisted coiling, i.e., flow diversion, however might not be deemed appropriate given the fact that anti-platelet agents would have been required henceforth, with treatment of the BAVM still pending. The effects of BAVM treatment on the natural history of proximal flow–related IAs is vaguely reported in the literature. Redekop and colleagues reported on only one (4.3%) patient with proximal flow–related IAs that disappeared following BAVM treatment, with four (17%) IAs becoming smaller, while IAs that arise on distal feeding arteries had a high probability of regressing when >50% reduction of the BAVM was attained (5). Alike, Eliava S. et al. reported one patient where the aneurysm spontaneously regressed after AVM treatment in a total of 205 aneurysms associated with BAVM (23).

Yet no solid conclusions can be drawn whether coexisting IAs and other BAVM characteristics are risk factors for subsequent hemorrhage (18, 24). Based on a literature review and on institutional experience, Flores and colleagues proposed that for unruptured BAVM with proximal flow–related IAs, treatment decision should be based on the IAs themselves (12). As such, bleeding risk should be considered, taking into account the IA's size, morphology, and location and age-specific risk factors as reported by the International Study of Unruptured Intracranial Aneurysms (ISUIA) (25, 26). Thus, given the size of both flow-related IAs, treatment might have been indicated with regard to potential rupture before the elimination of the BAVM. The same was true for the unrelated AComA. Given the history of aSAH, treatment toward endovascular coil embolization in the 4 mm IA was favored (21, 22). As for the unruptured BAVM itself, according to the ARUBA trial, the spontaneous annual risk of hemorrhage was noted to be 2.2% (13). Therefore, it remains controversial whether the size of the BAVM is a strong predictor for hemorrhage. While some studies reported an increased risk of hemorrhage in small BAVMs (<3 cm) (27, 28), others noted an increased risk of subsequent hemorrhage in larger BAVMs (>5 cm) (14, 29). With a SSM grade of ≤6 of this BAVM, surgical morbidity is acceptably low (30). We proceeded with partial intraoperative BAVM embolization, followed by surgical resection, with immediate control 3D-DSA revealing spontaneous regression of the two flow-related aneurysms. This finding was confirmed by follow-up MRA at six months and one-year follow-up. Potential flow reduction through feeding arteries and subsequent thrombosis and thus IAs regression following BAVM resection might have been the underlying mechanism (5, 6). Given the missing treatment guidelines the treatment risk should be carefully weighed against the natural history of the disease, which remains poorly understood (12, 31).

## Conclusions

Given the missing treatment guidelines and the still startling regression of flow-related aneurysms encountered on treatment of the associated BAVM, the surgical and interventional risk should be carefully weighed against the natural history of the disease, which remains poorly understood.

## Data Availability

The original contributions presented in the study are included in the article/Supplementary Material, further inquiries can be directed to the corresponding author/s.
